# Strengths, Struggles, and Strategies: How Adults with Serious Mental Illness Navigate Long-Term Romantic Relationships

**DOI:** 10.1007/s10597-024-01288-1

**Published:** 2024-05-07

**Authors:** Catherine H. Stein, Rachel A. Redondo, Sharon Simon, Zachary J. Silverman

**Affiliations:** Department of Psychology, Bowling Green State University, Bowling Green, OH 43403 USA

**Keywords:** Serious mental illness, Long-term romantic relationships, Qualitative methods, Romantic relationship strategies, Romantic relationship strengths

## Abstract

Married individuals and those in committed romantic relationships generally report having better mental health outcomes than their single or divorced counterparts. However, studies of romantic relationships for adults with mental illness have often ignored rewarding aspects of romantic relationships and have focused primarily on relationship difficulties. In this study, 23 adults with serious mental illness in long-term romantic relationships described their relationship strengths and struggles in small focus group discussions. Content analysis was used to characterize themes from participant accounts. Overall, participants described deep emotional bonds with their partners, a mutual willingness to work on their relationship, and good communication skills as relationship strengths. Mental health symptoms and internalized stigma were identified as major contributors to relationship struggles. Participants spontaneously identified intentional strategies that they used to navigate mental health challenges in their relationship that included self-directed, partner-directed, and couple-directed actions. Implications of findings for research and practice are discussed.

It is estimated that over 14 million adults in the United States have a serious mental illness that includes conditions such as schizophrenia, bipolar disorder, major depression, and personality disorders (National Institute of Mental Health, 2021). Recovery from serious mental illness is seen as a process through which individuals learn to improve their health and wellness and strive to live self-determined and satisfying lives (SAMHSA, 2012). Social connectedness and supportive relationships with family and friends have consistently been found to relate to mental health recovery (Cullen et al., 2017; Nasser & Overholser, 2005; Windell & Norman, 2013). However, relatively few studies have examined the nature of romantic relationships when one or both partners is living with serious mental illness (Mizock et al., 2019; White et al., 2021).

Finding a romantic partner can be challenging for adults with a serious mental health condition due to factors such as stigmatization, lack of resources, and mental health symptoms. Social stigma can foster discrimination and exclusion of adults with serious mental illness in a variety of life domains, including social interactions and romantic relationships (Budziszewska et al., 2020). For example, vignette studies suggest that adults’ reports of their willingness to engage in romantic relationships with an attractive partner significantly decreased upon learning that the person had a mental illness (Boysen, 2017; Boysen et al., 2019). Individuals with serious mental illness can also experience internalized or self-stigma where they inculcate feelings of devaluation, shame, and withdrawal by applying negative stereotypes about mental illness to themselves (Corrigan & Rao, 2012; Van Brakel, 2006). Psychiatric symptoms can negatively impact adults’ employment opportunities, autonomy in living arrangements, and financial security which can limit meeting and dating potential romantic partners (Angell & Test, 2002; Pillay et al., 2018). Psychiatric symptoms can also impact interpersonal and cognitive skills that can lead to fewer socializing opportunities (Pillay et al., 2018).

In general, married individuals and those in committed romantic relationships report higher overall levels of mental health than their single or divorced counterparts, including people who are coping with serious mental illness (Braithwaite & Holt-Lunstand, 2017; Haro et al., 2008). Studies of the influence of romantic relationships for adults with psychotic disorders found that marriage or having been married was associated with better symptom outcomes and longer remission times as compared with adults who were single or never married (Jorgensen & Aagaard, 1988; Lang et al., 2019). However, adults with serious mental illness generally report poorer overall relationship quality than adults without mental illness (Aggarwal et al., 2019; Pankiewicz et al., 2012; Segrin et al., 2003). In addition, higher levels of internalized stigma were generally found to be related to lower perceived assertiveness and lower levels of satisfaction in romantic relationships for individuals diagnosed with schizophrenia and bipolar disorder (Sarisoy et al., 2013).

Overall, existing studies focused on specific characteristics of romantic relationships among adults with mental illness highlight the disruptive or dysfunctional influence of mental illness on relationships. In a study by Sharabi et al. (2016), a sample of 135 couples diagnosed with various types of depression responded to an open-ended survey question about how feelings of sadness or depression affected their romantic relationship. Eight negative effects of depression on romantic relationships were identified that included emotional toll, sexual intimacy issues, communication problems, social isolation, lack of energy/motivation, dependency, lack of understanding, and uncertainty. Study participants only identified one positive effect, enhanced intimacy, as a result of having one or both partners with depression. Similarly, a qualitative study focused on dating among 20 women with serious mental illness who were currently or had at one time been in a romantic relationship identified pathologizing problems, symptom interference, social or self-stigma, deprioritizing dating, and finding a partner with the same perceived level of psychosocial functioning as challenges to dating and maintaining romantic relationships (Mizock et al., 2019). Research with a sample of 10 couples where one partner had a serious mental illness found that couples experienced changes to social roles, emotional upheaval and interpersonal distance in the relationship, and a sense of failure from the partner with mental illness in accomplishing expected social role activities within the relationship (Mokoena et al., 2019).

Although some prior research has examined types of coping among adults who had a partner with a mental illness (Acevedo Callejas & Thompson, 2017), few studies have focused on how adults who are currently in long-term relationships describe their relationships or identify strategies that they find meaningful in facing mental health challenges. Studies are also lacking that examine potentially rewarding as well as challenging aspects of long-term romantic relationships for adults with mental illness. Research focused on long-term romantic relationships from the perspective of adults with mental illness is fundamental to the development of evidence-based social relationship interventions. Strength-based studies of long-term romantic relationships have implications for how people with serious mental health conditions view themselves and are viewed by mental health care professionals.

The present study examined the narrative accounts of adults with serious mental illness in describing characteristics of their current long-term romantic relationships. Specifically, we asked adults with mental illness to describe their strengths and struggles in their ongoing romantic relationships to allow study participants to reflect upon their experiences in the context of small focus groups. To help fill a gap in existing literature, the present study purposefully focused on the perspectives of adults with mental illness who reported currently being in long-term romantic relationships. The primary goals of the present study were to: (1) examine ways that adults with mental illness describe navigating their long-term romantic relationships that included potentially rewarding and challenging relationship aspects and (2) describe the role of mental illness in romantic partner relationships from the perspective of the partner with a psychiatric diagnosis.

## Methods

### Participants

The sample consisted of 23 adults living in the United States who reported that they had a serious mental illness and had been in a committed romantic relationship for one year or longer. Demographic characteristics for the sample can be found in Table 1. A total of 14 adults identified as female, five identified as male, and four identified as non-binary. The mean age of the sample was 31.6 years (*SD* = 11.2), and the average length of the current romantic relationship reported by the sample was 6.2 years (*SD* = 5.7). A total of 39.1% of participants (*n* = 9) reported that they were married and 86.9% reported that they were cohabiting with their partner. Sample participants identified as White (73.9%; *n* = 17), a Person of Color (17.4%; *n* = 4), or Multiracial (8.7%; *n* = 2) and a majority of the sample (86.9%; *n* = 20) reported that they were currently in a heterosexual relationship. In terms of educational status, almost half (47.8%, *n* = 11) of the sample reported that they had graduated high school, attended some college or had earned a two-year degree, about 30% (*n* = 7) reported holding a bachelor’s degree, and about 22% (*n* = 5) reported earning a post-graduate or professional degree. Most of the sample (60.8%; *n* = 14) reported being employed either full-time or part-time.

**Table 1:** Sample characteristics (*N* = 23)

Characteristics	Number (%)	Characteristics	Number (%)
Gender		Age	
Female	14 (60.9)	*M =* 31.6 years *(SD =* 11.2)	
Male	5 (21.7)		
Nonbinary	4 (17.4)	Length of Relationship with Partner	
		*M =* 6.2 years *(SD =* 5.7)	
Race/Ethnicity			
White	17 (73.9)	Primary Psychiatric Diagnosis	
BIPOC	4 (17.4)	Mood Disorder	5 (21.7)
Multiracial	2 (8.7)	Dissociative Disorder	5 (21.7)
		Schizophrenia-related disorder	4 (17.4)
Education		Borderline personality disorder	3 (13.0)
High school diploma/GED	5 (21.7)	Posttraumatic stress disorder	3 (13.0)
Some college/Associates	6 (26.1)	Anxiety or OCD-related disorder	2 (8.7)
Bachelors	7 (30.4)	Eating disorder	1 (4.3)
Postgraduate degree	5 (21.7)		
		Number of Psychiatric Hospitalizations	
Employment Status		0	6 (26.1)
Full-time	10 (43.5)	1–2	8 (34.8)
Part-time	4 (17.4)	≥ 3	9 (39.1)
Not employed	8 (34.8)		
Student	1 (4.3)	Receiving Treatment for Mental Health	
		Yes	21 (91.3)
Annual Income		No	2 (8.7)
< $30,000	11 (47.8)		
$30,000–70,000	7 (30.4)	Taking Medication for Mental Health	
$70,000-110,000	4 (17.4)	Yes	18 (78.3)
> $110,000	1 (4.3)	No	5 (21.7)
Marital Status ^a^		Partner Has Psychiatric Diagnosis	
Not married	13 (56.5)	Yes	15 (65.2)
Married	9 (39.1)	No	8 (34.8)
Living Situation			
Co-habiting	20 (86.9)		
Separate Household	3 (13.0)		
Monogamous Relationship ^b^			
Yes	20 (86.9)		
No	3 (13.0)		
Number of Children			
0	19 (82.6)		
1	1 (4.3)		
2	3 (13.0)		

^a^ One participant did not report marital status

^b^ Participants in polyamorous relationships described relationship with primary partner only

Based on participant reports, the types of primary psychiatric diagnoses for the sample included mood disorders (21.7%; *n* = 5), trauma or anxiety-related disorders (21.7%; *n* = 5), dissociative disorders (21.7%; *n* = 5), schizophrenia-related disorders (17.4%; *n* = 4), borderline personality disorders (13%; *n* = 3) and eating disorders (4.3%; *n* = 1). A total of 11 participants reported that they had initially met their partner online and 12 participants reported that they had initially met their partner in-person. A majority (65.2%; *n* = 15) of romantic partners were identified as male and participants identified partners as White (95.7%; *n* = 22) or a Person of Color (4.3%; *n* = 1). Sample participants reported that 65.2% (*n* = 15) of their partners also had a diagnosed mental health condition.

### Procedure

The research study was approved by the Institutional Review Board of Bowling Green State University. To be eligible to take part in the research, participants needed to live in the United States, be 18 years or older, have a diagnosed mental health condition, and be in a romantic relationship for one year or longer. To inform individuals about the study, a brief description of the research and requirements for participation was posted on a variety of social media sites that hosted support groups, forums, or chats focused on dating and/or mental health issues (e.g., dating/mental health subreddits on reddit.com; Facebook mental health support groups; specialized dating sites). Researchers contacted website administrators when needed to obtain permission to post a description of the study on their website. Individuals interested in the study completed a brief phone screen to receive more information about the study and to determine eligibility. Of the 69 adults who responded to posts about the research on social media, 29 individuals completed a phone screen, 28 of those people met the eligibility criteria, and 23 individuals took part in focus group interviews. Individuals who completed a phone screen for the study but did not participate in the research did not significantly differ from study participants on basic demographic characteristics (i.e., age, gender, diagnosis, relationship characteristics, social media use).

Eligible individuals participated in small focus groups conducted online via a secure video conferencing platform that lasted approximately 60 to 90 min. Focus groups ranged in size from three to six participants and were facilitated by pairs of doctoral students in clinical psychology who had extensive experience with the interview protocol. The size and composition of focus groups were based on how participants had met their partner (i.e., online or in-person) and participants’ availability to meet at specific dates and times. Three of the six focus groups consisted of participants who met their partners online (*n* = 11) and three focus groups were composed of participants who met their partners in-person (*n* = 12). We organized groups based on the similarity of how participants met their partners in an effort to facilitate rapport among participants within focus groups. As a token of appreciation, each participant received a $10 gift card for their participation in the research. The six focus groups were videotaped, and audio content was transcribed verbatim. Participant ID numbers were used in data analysis and pseudonyms are used in the presentation of study findings to protect confidentiality of participants.

### Measures

#### Dating Focus Group Interview Protocol

A semi-structured focus group interview protocol was developed for the present study to enable participants to share their experiences on a variety of topics. At the start of focus group interviews, participants were asked to provide the group with some background about their romantic relationship that included the first name of their partner, how they met, and the length of their relationship. During the focus group sessions, participants were asked several broad, open-ended questions about their romantic relationships designed to facilitate interactions among group members. The interview protocol included questions related to the nature of participants’ relationship with their partner, and their views of strengths and struggles in their romantic relationship. Focus group protocol included initial prompts such as “Tell us about yourself and your relationship with your partner” and “What do you consider to be the strengths and struggles of your relationship?”

### Data Analysis

Focus group interviews were transcribed verbatim and examined using content analysis techniques (Miles et al., 2018). Atlas.ti qualitative software (Hwang, 2008) was used to assist in data organization and management. A seven-member research team repeatedly read focus group transcripts and engaged in an iterative process of identification, comparison, and refinement to articulate emergent concept themes and overarching concept categories from participant accounts. Ideas and themes about the nature of participants’ relationships with partners were identified and compared within and across transcripts as a first step in data analysis. Initial codes were generated for meaning units that consisted of words, sentences, or larger portions of verbatim text. An initial codebook was developed with operational definitions of codes and representative quotes. In a next step in the coding process, research team members grouped, modified, and refined initial coding categories to generate emergent themes. Researchers met regularly to discuss, interpret, and refine themes using transcript data to support data analytic claims. Discrepancies among researchers in coding were further reviewed by subsets of research team members and were resolved by group discussion and consensus by the entire research team (Edwards et al., 2004). An iterative process of selective coding was undertaken to connect and codify themes into overarching concept categories.

### Researcher Positionality

In the research context, positionality requires that researchers situate themselves in each aspect of the research process through reflexivity to consider social, racial, and political contexts and personal identities that may directly or indirectly impact the research (Holmes, 2020). In the present study, a majority of research team members identified as White (6 White and 1 Multiracial team member), female (5 female, 1 male, and 1 nonbinary team member), and married or currently in a committed romantic relationship (4 team members in a relationship and 3 team members not in a romantic relationship). To potentially align with participants, we ensured that at least one of the two doctoral student co-facilitators for each focus group was currently in a committed romantic relationship. During focus group introductions, co-facilitators described their relationship status and background along with participants. Some co-facilitators who identified as having a mental health condition chose to disclose their mental health status to participants in focus groups. Focus group co-facilitators who had a mental health condition and chose to disclose it in focus group introductions were motivated by authenticity in the research context (Manning, 1997). Throughout the process of data analysis, research members discussed their backgrounds, perspectives, and experiences relevant to the study to make assumptions that may impact data interpretations more explicit.

## Results

Focus group participants were asked to describe strengths and struggles that they currently experience in their relationship with their partner. Relationship strengths were defined as positive qualities that participants identified as basic to the success of their relationship with their partner. In contrast, relationship struggles were defined as aspects that participants viewed as problematic, difficult, or in need of improvement. Of the 23 adults in the present study, 18 (78.3%) identified at least one strength in their relationship with their partner, and 18 (78.3%) identified at least one aspect of their partner relationship as a struggle. Relationship strengths and struggles themes described by participants are summarized in Table 2.

**Table 2:** Relationship strengths and struggles themes, operational definitions, and representative quotes (*N* = 18)

Category	Theme	Definition	*n* (%)	Quote
Strengths				
	Deep emotional bond with their partner	Couples create a strong union, love, friendship and understanding.	12 (66.7%)	“… I have someone [my partner] in my life who understands me, and who knows what it is like, and who is a safe place for me…He is the only person that I have ever met who understands me so well.” --Elaine
	Mutual willingness to work on the relationship	Couples’ motivation and mutual dedication to work to improve their relationship.	9 (50.0%)	“… We [my partner and I] have a strong belief that it is not enough just to say you are sorry…You have to do something to make it better. You have to actively try to fix it.” --Jennifer
	Good communication skills	Couples active use of good communication skills.	8 (44.4%)	“I know communication has been very important to us [my partner and me], because we have seen in other relationships how assumptions and mind reading… can really lead to trouble.” --Charles
	Personal qualities that foster compatibility	Personal characteristics attributed to getting along or feeling a sense of connection in the relationship.	17 (94.4%)	“He is so fun. He is like the funnest person you’ll ever meet.” --Hannah
Struggles	Challenges navigating mental health symptoms	Difficulties with mental health symptoms, including having difficulties identifying/communicating symptoms and developing boundaries.	13 (72.2%)	“I am definitely able to hide stuff from him [my partner], which is not always good when I am feeling crappy or when I am feeling unwell.” --Dante
	Dealing with internalized stigma	Internalized stigmatization of mental illness or other negative self-beliefs that impact the relationship.	10 (55.5%)	“He [my partner] says, ‘I love you,’ and it has been almost three years, and I am finally starting to believe him.” --Brianna
	Personal qualities that foster incompatibility	Personal characteristics attributed to not getting along or hindering a sense of connection in the relationship.	10 (55.5%)	“Differences [between my partner and me] in terms of him being super neat and athletic and liking to cook. And I am not any of those things.” -Julie

### Relationship Strengths

Over half of participants (*n* = 12/18; 66.7%) described having a *deep emotional bond with their partner* where they and their partner understood each other on an intimate, emotional level. These adults described experiences of emotional closeness, safety, love, friendship, and admiration for their partners. The sentiments of participants reflect affection and intimacy as foundational aspects of their romantic relationships. As James simply states: “We [my partner and I] just need to be together. I need her in my life, and I feel that she needs me in her life.” Some participants expressed their emotional connections as a couple despite having to deal with serious mental illness in the relationship. Brianna recalled her partner’s statement with love and gratitude: He [my partner] once said to me, “I would always put up with the bad days, because the good days are just so worth it.”

A *mutual willingness to work on the relationship* was also identified by participants (*n* = 9; 50%) as a basic relationship strength. These adults described their own and their partner’s ability to admit and correct their mistakes for the good of their relationship. As Luke states: “Admitting when I make a mistake and taking action to make amends…we [my partner and I] definitely try to achieve that to avoid resentment.” Participant accounts included a stated commitment to building skills that will positively impact the relationship, a need to be honest with each other to build trust, a willingness to tolerate each other’s behaviors, and a dedication to working together to solve relationship problems. As Emily describes: “We’re very honest with each other [my partner and I], the one thing that’s gotten us through everything is honesty…it builds trust…you always know someone has your back and is watching the way that you’re reacting to things [events and/or circumstances that occur in daily life], and you watch the way that they’re reacting to things, and you trust that.”

In addition, eight (44.4%) participants’ accounts described the importance of *good communication skills* as a relationship strength that included listening, trying not to make assumptions, and avoiding shouting or calling their partner names. These adults also discussed how being committed to having difficult conversations and being willing and able to express uncomfortable thoughts and feelings led to more effective communication in their relationship. Charles explains: “Communication has been very important to us [my partner and me]…because we’ve seen in other relationships how assumptions and mind reading can really lead to trouble.” Overall, participants believed that good communication is necessary in all romantic relationships but that it is especially important in romantic relationships where one or both partners are coping with mental health issues.

A majority of participants (*n* = 17; 94.4%) described themselves or their partner as having *personal qualities that foster compatibility* in their relationship that included being patient, calm or well-balanced, being supportive, accepting, or non-judgmental, being good with logistical tasks, or being good with expressing emotions. In describing her own positive qualities, Elaine explains: “I think that I can be really gentle and really warm and really loving and really supportive. And I try really hard not to tear him [my partner] down, to be kind and to be considerate and to be open to meeting his needs.”

### Relationship Struggles

A majority (*n* = 13; 72.2%) of adults in the present study focused on *challenges navigating mental health symptoms* as a major source of relationship struggles. Some participants described having problems articulating their own mental health symptoms or understanding one another’s mental health condition when both partners had a psychiatric diagnosis. In some situations, participants described that they tried to hide their mental health symptoms from their partners. Other participants described initially lacking awareness or understanding of their mental health condition, which hindered their ability to seek help, and ultimately, negatively impacted their relationship. As James shares, “We [my partner and I] always knew something was off, but I did not really seek out help for it [my mental health issues] until we had this big blow out and actually ended up breaking up for a little while.” Participants often expressed difficulties identifying external or internal cues (i.e., triggers) that could potentially activate their emotions, which would sometimes lead to partners unintentionally exacerbating their mental health difficulties.

Participants discussed how their mental health symptoms, such as being unable to focus on their partner’s words, sometimes negatively impacted the relationship. Participants also described some unique challenges when both members of the couple were coping with serious mental health conditions since even partners with the same psychiatric diagnosis often manifested their symptoms in different ways. Elizabeth’s description of differences between herself and her partner’s manifestation of bipolar disorder symptoms is particularly poignant: “When he [my partner] found out that he was bipolar, he smashed out somebody’s windows and went to jail. When I found out I was bipolar…I tried to kill myself. He didn’t really understand why I would want to die when I’m manic because he is just destructive when he’s manic.”

Adults in the present study (*n =* 10; 55.5%) also described *dealing with internalized stigma* as a source of relationship struggle. These participants said that that they felt like a burden to their partner or are not an ideal partner due to their mental health difficulties. Some participants described how their partner had to serve as a caregiver due to their mental health symptoms. For example, Emily states, “… there have been times that he [my partner] has felt like he has had to be more of that [caregiver] role… I really needed his help and I actually needed someone to care for me.” Some participants also shared that they continue to think that their relationship might end at any moment due to their mental health issues. Other participants reported that it took a long time for them to believe that their partner loved them, even after repeated declarations of love by their partner. These participants expressed the feeling that their mental illness made them unlovable.

About half of the sample (*n* = 10; 55.5%) identified *personal qualities that foster incompatibility* in their relationship that included differences in the ways that each partner expresses love and affection, dissimilar family backgrounds, and differences in their sex drive and in their overall energy levels. Alison describes how differences in habits and energy levels between herself and her partner can lead to distress: “… my boyfriend…is super motivated and super athletic, he does so much…I do not exercise. He is not going to want to stick with me because I am gaining so much weight.” Qualities such as jealousy or expressing anger were also identified as participant or partner qualities that caused relationship struggles.

### Strategies for Navigating Mental Health Challenges

Adults in the present study spontaneously described how they and their partner dealt with mental health challenges that arose in their relationship. In participant accounts, we identified strategies as intentional techniques that participants reported using to navigate mental health challenges experienced by participants and/or their partners. All 23 participants in the present study described the use of at least one mental health strategy in the context of their romantic relationships. These strategies provide a nuanced perspective on ways that participants recognize and intentionally attempt to deal with mental health issues in their romantic relationships. Overarching theme categories from participant accounts reflect intentional mental health strategies participants used to address their own mental health challenges (*self-focused mental health strategies*), strategies participants used to deal with their own mental health challenges that involved help from their partner (*partner-involved mental health strategies*) and strategies used by both partners jointly to deal with mental health challenges thought to impact their romantic relationship (*couple-involved mental health strategies*). Table 3 provides the operational definitions for each theme in these three overarching categories, the frequency of themes reported, and representative quotes.

**Table 3:** Strategies for navigating mental health themes, operational definitions, and representative quotes (*N* = 23)

Category	Theme	Definition	*n* (%)	Quote
Self-focused	Attending to my own mental health needs.	Active engagement in psychotherapy, use of mental health medication, and/or reliance on informal supports to take care of individual mental health needs.	8 (34.8%)	“…I want to continue to work hard and work through those triggers and do that in therapy… I do not want to be triggered by my partner sneezing.” --Riley
Partner-involved	Talking about current mental health symptoms or experiences with partner.	Unidirectional communication by participant to partner about current mental health symptoms or needs related to symptoms.	9 (39.1%)	“… I have gotten comfortable with… [communicating to my partner that] I am not getting out of bed today when I need a break” --Brianna
	Using partner as a reality check.	Relying on partner as a grounding force or reality test related to mental health symptoms such as catastrophic or delusional thinking.	9 (39.1.%)	“… Every now and then I will get a reality check [about my delusional thinking]…from him [my partner]… I will say, ‘This is just a coincidence that this happened. This is not a sign of the apocalypse or anything, right?’” --Kayla
	Partner checking in about my mental health symptoms.	Partner noticing participant’s mental state and checking in to see how they are doing regarding mental health issues.	7 (30.4%)	“My wife has gotten better about… picking up on things …If I am depressed and isolating myself, she will always… reach out and [say], ‘Are you okay? Is there anything I can do?’” --James
Couple-involved	Mutual dialogue between partners about mental health issues.	Both partners engaging in conversations about mental health issues.	13 (56.5%)	“… To be able to say to him [my partner], ‘Hey, this thing does not make me feel good.’ … And for him… [to say], ‘I noticed you were quiet…’ Our communication is really the foundation of our relationship…” --Luna
	Balancing partners’ mental health needs.	Striving to balance mental health needs of both partners in the relationship.	6 (26.1%)	“… if I am really emotional and I need a lot right now…we are dealing with me, and what I need…whereas my partner’s needs take a back seat. And sometimes its switched… I put my issues aside so that we can take care of him [my partner].” --Elaine

*Self-focused mental health strategies* (*n* = 8/23; 34.8%) centered on participants as they described *attending to my own mental health needs*. These strategies included ways that participants sought out professional help such as trying to actively engage in psychotherapy, and/or working to adhere to a psychiatric medication regime. Participants also described using informal sources of help such as seeking support for their mental health issues from family members or friends. These adults felt that seeking formal and informal support to attend to their own mental health needs were ways they could positively impact their relationship with their romantic partner.

*Partner-involved mental health strategies* focus on ways that participants described relying on their partners for help in navigating their mental health challenges. Some participants (*n* = 9; 39.1%) described *talking about current mental health symptoms or experiences with my partner* as one type of partner-involved strategy. These conversations consisted of unidirectional communication of current mental health symptoms by participants to their partners or needs related to their symptoms. Some participants described how they have regular conversations with their partner to discuss what they are learning in therapy, how they are currently doing with their mental health, and/or how their partner can help them with specific mental health challenges. Some participants reported having conversations with their partners about unexpected or sudden challenges to their mental health. For example, Jamie states, “… when they [my triggers] come up…we [my partner and I] talk about it afterwards.”

*Partner as a reality check* is another partner-involved mental health strategy that described participants’ accounts (*n* = 9; 39.1%) of relying on a partner as a touchstone or grounding force when experiencing mental health symptoms. Some participants reported that their partner uses specific grounding techniques, such as reminding them that they are ‘real’ or ‘present,’ to help them cope with depersonalization or derealization. Other participants described how they ask for feedback about their reactions to a variety of situations. For example, Emily who is in a romantic relationship where both partners have a psychiatric diagnosis states, …we [my partner and I] can look at each other and [say], ‘Hey, am I overreacting?’ And [the other person] will [say], ‘Yeah, you are.’” Participants (*n* = 7; 30.4%) also described the importance of *‘checking in’ about mental health symptoms* as a partner-involved strategy for navigating mental health challenges. Participant accounts described ways that their partners recognize participants’ mental health symptoms and ask about their needs. For example, Samantha describes that their partner sometimes inquires: “… ‘Hey, I feel like you [Samantha] are iffy right now. Is there something I can do?’”.

Two themes from participant accounts reflect c*ouple-involved mental health strategies* where participants report that they and their partner work together to address mental health issues thought to impact the relationship. Most participants (*n* = 13; 56.5%) discussed having an *open dialogue about mental health challenges in their relationship* where both partners mutually engage in conversations focused on mental health issues. *Balancing partners’ mental health needs* reflects participants’ accounts (*n* = 6; 26.1%) of ways that couples prioritize and address both partners’ mental health needs in their relationships. These adults reported how they adapt when one partner is experiencing increased psychiatric symptoms by taking over the partner’s daily tasks such as cooking or grocery shopping or altering the couple’s overall roles or routine. Participants also discussed being able to help their partner at times when it is difficult to help themselves. As Samantha explains: “Taking on the caregiver role gives you something you can be powerful about, when you can’t handle your own [problems], you can do for another [person]. I can help my partner.” It is noteworthy that couple-involved mental health strategies were described by both participants who reported having a partner with mental illness and by participants who did not report having a partner with a psychiatric diagnosis. Participants who described using couple-involved strategies often emphasized the importance of dealing with mental health issues for both partners in romantic relationships.

## Discussion

Using focus group methods, the present study examined the accounts of 23 adults in long-term romantic relationships who self-identified as having a serious mental illness. Research findings characterize adults’ accounts of both positive and challenging aspects of their romantic relationships. As importantly, the present study highlights the intentional strategies that these adults reported that they use to deal with mental health challenges within the context of their romantic relationships.

Present study findings underscore the reality of persistent psychiatric symptoms in the lives of these adults and suggest various ways that symptoms shape romantic relationships. Consistent with previous research, mental health symptoms were identified as a major contributor to relationship struggles as both individuals and their partners were confronted with changes in mood, thinking, and behavior related to psychiatric symptoms (Sharabi et al., 2016). Participants’ accounts also contribute important insights into the range of challenges attributed to psychiatric symptoms not identified in previous research. Study participants identified consequences of some mental health conditions such as problems with memory and concentration that negatively impacted interactions with their partner. Participants also noted an initial lack of awareness or understanding of their mental health condition that impacted their relationship with their partner. The present study highlights the critical need for psychoeducation to help individuals with mental illness and their loved ones recognize and manage psychiatric symptoms (Motlova et al., 2017).

Some adults in the present study identified internalized stigma as an ongoing relationship struggle that perpetuated feelings of being unworthy of love. Present results are consistent with a study by Sarisoy and colleagues (2013) that found that adults with mental illness who reported internalized stigma also generally reported greater concerns about romantic relationships and less overall relationship satisfaction than did their counterparts who did not report internalized stigma. Unfortunately, a majority of participants in the Sarisoy et al. (2013) study were not married or in a current romantic relationship. Therefore, it is unclear what frame of reference for romantic relationships participants in the Sarisoy et al. (2013) study were using which limits interpretation of study results. However, present study findings provide support for the importance of internalized stigma for adults currently in long-term romantic relationships. Future research is needed to replicate and extend present study findings on internalized stigma in romantic relationships and to examine ways that romantic partners may assist adults in developing resistance to self-stigmatizing attitudes (Jahn et al., 2020).

Adults described confronting their serious mental health challenges within the context of long-term romantic relationships that they felt nurtured love, caring, and connectedness. Participants described relationship strengths in terms of deep emotional bonds that they have with their partner, good communication skills, and a dedication to making their relationship work. Study participants described feelings and actions that emphasized investment, interdependence, and a long-term orientation towards the relationship and towards their partner that are characteristic of relationship commitment (Rusbult & Buunk, 1993). Contextualizing romantic relationships in terms of relationship commitment may be particularly important in understanding how couples deal with challenging life circumstances associated with serious mental illness (Gamarel et al., 2019). Similar to a previous study of spouses of people with mental illness (Lawn & McMahon, 2014), adults in the present study described their efforts to nurture intimacy and emotional commitment, despite challenges they face as a result of serious mental illness. The present study underscores the supportive nature of romantic relationships as described by participants and the importance of research that examines both perceived rewards and challenges of long-term romantic relationships. Present study findings can motivate future research that examines similarities and differences in perceived social support from romantic partners, family members, and friends in facilitating mental health recovery for adults with serious mental illness.

Unlike prior research, the present study describes intentional strategies identified by participants to specifically deal with mental health challenges in their relationships. Mental health strategies discussed by participants ranged from self-focused approaches such as attending therapy and taking psychotropic medication, to partner-involved strategies such relying on their partner as a trusted other to serve as a reality check for their symptoms. Adults’ accounts also included couple-involved mental health strategies used by both members of the couple such as engaging in mutual dialogue between partners about mental illness and working to balance both partners’ mental health needs. Participants’ accounts reflect an active stance in facing mental health issues both as individuals and together with their partners. Similar to research with couples facing physical illness, adults in the present study seemed to cultivate the positive aspects of their relationship in spite of mental illness, which may serve to bring partners closer together (Mahrer-Imhoff et al., 2007). More research is needed that focuses on couples who face serious mental health conditions to understand individual, social, and relationship factors that positively shape romantic relationship maintenance and growth and mental health recovery.

### Study Limitations and Implications for Research and Practice

Although present study findings are thought provoking, the research is limited in several respects. Study findings are based on a relatively small, non-random sample of mostly White female participants. Descriptions of adults’ psychiatric diagnoses were based on participant self-report. Individuals learned about the research from online venues which may reflect a general willingness to share their perspective on mental health issues with others. The size and composition of focus groups may have shaped the nature of discussion among participants in unknown ways. The sample was heterogeneous with respect to self-reported age, length of romantic relationship, and psychiatric diagnosis. Although this sample heterogeneity captures the experiences of a relatively diverse group of adults, such sample heterogeneity may mask underlying differences in participants’ accounts as a function of phase of the life course. The potential impact of research design elements such as participant selection, sample characteristics, and focus group methods require further investigation to identify factors most relevant to studying long-term romantic relationships among adults with mental illness. Future research using larger samples of adults with specific types of psychiatric diagnoses is also needed to contextualize present study findings.

The majority of adults in the present study reported that their current romantic partner also had a psychiatric diagnosis. For a variety of reasons, we intentionally limited the present study to focus on the lived experience of adults with mental illness without regard for their partners’ mental health status. Epidemiological evidence suggests that adults with a psychiatric condition are more likely to marry people who also have a psychiatric diagnosis than would be expected by chance or than individuals with non-psychiatric conditions (Nordsletten et al., 2016). Future research is needed to examine the nature of romantic relationships when both partners have a mental illness to understand the unique benefits and challenges that may accompany these relationships for both members of the couple. Studies that focus on the couple as the unit of analysis are needed to understand the mutual influence of both partners’ perceptions and behaviors in navigating romantic relationships within the context of mental illness.

We purposefully focused our research on adults who were navigating a long-term romantic relationship so that participants could speak directly to experiences with their current partner. Researchers can amplify and legitimate the experiences of marginalized groups through the methodological choices that characterize their research (Stein & Mankowski, 2004). The present study helps to challenge stigmatizing attitudes about adults with mental illness by examining both strengths and struggles in the accounts of adults who are currently engaged in long-term romantic relationships. Our research design and sample selection remind researchers and practitioners that, despite potential barriers, adults with mental illness can and do maintain long-term romantic relationships. Our findings suggest commonalities between romantic relationship strengths identified by adults with mental illness such as the importance of communication and those strengths identified for successful romantic relationships in general (Yoo et al., 2014). Research grounded in the lived experience of adults with mental illness who are engaged in long-term romantic relationships is essential for the development of evidence-based relationship interventions for people with mental health conditions. Further research on specific strategies used in romantic relationships by adults with mental illness can inform community mental health programs focused on mental health recovery. The present study serves as an important first step for future strengths-based research on long-term romantic relationships for adults with serious mental illness.
